# Performance Assessment of Microfiltration Membranes for Backwash Water Reuse from Sand Filters

**DOI:** 10.3390/membranes16050169

**Published:** 2026-05-04

**Authors:** Małgorzata Wolska, Małgorzata Kabsch-Korbutowicz, Fausto A. Canales, Javier Carpintero, Halina Urbańska-Kozłowska

**Affiliations:** 1 Faculty of Environmental Engineering, Wroclaw University of Science and Technology, 27 Wybrzeże Wyspiańskiego st., 50-370 Wrocław, Poland; malgorzata.kabsch-korbutowicz@pwr.edu.pl; 2 Civil Engineering Program, Faculty of Engineering, Universidad Militar Nueva Granada, Campus Cajica, Cajica 250247, Colombia; fausto.canales@unimilitar.edu.co; 3 Department of Civil and Environmental Engineering, Faculty of Engineering, Universidad de la Costa, Calle 58 #55-66, Barranquilla 080002, Colombia; 4 Wroclaw Municipal Water and Sewage Company, Na Grobli 14/16, 50-421 Wrocław, Poland; halina.urbanska@mpwik.wroc.pl

**Keywords:** psychrophilic bacteria, backwash water, cost analysis, microfiltration, permeability

## Abstract

This study assessed the performance of three microfiltration (MF) membrane modules: M1 (spiral, polyvinylidene fluoride), M2 (capillary, polypropylene), and M3 (capillary, α-Alumina) in treating backwash water from utility-scale surface water (SW) and infiltration water (IW) plants, each with a capacity of approximately 100,000 m^3^/day. Considering 168 h (one-week) filtration cycles, the membranes were evaluated for permeate flux, turbidity removal, dissolved organic carbon (DOC) removal, and reduction in total number of microorganisms (TNM). In contrast to most previous studies that have primarily examined surface water sources under laboratory conditions, this research contributes to the literature by evaluating membrane performance using actual backwash water from both SW and IW treatment plants. The comparative assessment of three structurally and materially distinct membrane modules under identical flow-through conditions yields new insights into the trade-offs among hydraulic performance, contaminant removal, and treatment cost. Logarithmic models fitted to permeate flux data yielded determination coefficients (R^2^) ranging from 0.60 to 0.99, supporting the prediction of early-stage performance. No consistent trend in flux decline was observed, mainly due to fluctuations in the water bacterial load. With a median TNM removal efficiency of 97%, M1 outperformed M2 and M3 (84% and 70%, respectively) in terms of microorganism removal. The effectiveness of DOC removal generally depended on the type of backwash water; the highest efficiency was observed for M2 in the case of backwash from IW treatment and for M1 in the case of backwash from SW treatment. M2 provided the highest permeate flux rates, regardless of water type or operational limitations. The ceramic membrane (M3) exhibited the greatest variability in hydraulic performance and removal efficiency, depending on the type of backwash water. A simplified cost analysis over two filtration cycles found that treatment costs were generally higher for SW backwash, with differences reaching up to 90% between membrane-water type combinations. Although treatment costs are higher than those for raw water treatment, the increasing water scarcity makes it a potential additional source of safe water.

## 1. Introduction

The increasing global water deficit has underscored the need to implement a circular water economy, regardless of the specific use of water. For perspective, global water withdrawal increased from 0.67 to 3.79 trillion m^3^/year between 1900 and 2000 and is projected to rise by 55% by 2050 [1]. This pressure on resources also impacts water supply companies responsible for collecting and treating water intended for consumption [2]. As a result, research worldwide is being conducted to minimize water losses during treatment and distribution [3,4,5]. In the case of drinking water treatment, backwash water is a significant source of water loss in drinking water treatment plants [6]. This backwash can account for between 5% and 20% of the clean treated drinking water when using sand filters [4,7,8]. Given the sizable volumes generated, the reuse of backwash water is a viable strategy to reduce resource demand; however, it requires pre-treatment before being reintroduced into the water treatment system due to its contamination [5,9].

Regardless of the type of water being treated or the technological system used in the plants, backwash water typically contains high concentrations of suspended solids and exhibits microbiological contamination, including pathogenic and indicator microorganisms [10]. The choice of treatment methods for backwash water depends on its composition and results from pilot studies. These studies ensure that the level of contamination is reduced to match, or improve, the quality of the source water, especially if the treated backwash water is to be recirculated to points other than the beginning of the technological system, thus ensuring that treated backwash water does not impair the operating systems’ performance or threaten water consumers [11,12].

The composition of backwash water depends on the type of water being treated and the pollutants it contains. Surface water (SW) is most commonly used for drinking purposes, especially in large urban areas. However, due to the deteriorating quality of abstracted surface waters, infiltration water (IW) is increasingly being used either as a preliminary treatment of SW or to augment groundwater resources [13]. Due to partial purification in the soil, IW contains significantly lower amounts of organic substances and higher concentrations of mineral substances [14].

The infiltration method affects the concentration of pollutants in the infiltrated water and, consequently, the composition of the backwash water generated during treatment. Regardless of the infiltration method, infiltrated water and backwash water are characterized by the presence of microorganisms, including pathogenic ones, and high turbidity [15]. Thus, turbidity and the number of microorganisms in backwash water can serve as key indicators of the effectiveness of backwash water treatment for both SW and IW.

Microfiltration (MF) effectively removes suspended solids and microorganisms, making it a viable solution to improve backwash water quality, especially in systems facing challenges from infiltration-derived contaminants. Studies on membrane aging in full-scale potable reuse facilities indicate the need to optimize backwashing frequency to maintain filtration efficiency and minimize fouling [16]. There is limited information on the application of MF for treating backwash water under continuous-flow conditions in full-scale water treatment facilities. Existing studies on MF-treated backwash water have primarily focused on SW sources. For instance, Mueller et al. [17] applied ceramic membranes of different pore sizes (α-Al_2_O_3_ MF at 0.2 µm and SiC MF at 0.5 µm) in a pilot-scale setup for treating backwash water from a dam water treatment plant, achieving approximately 50% removal of humic substances, with fluxes ranging from 102 to 280 L/m^2^·h in cross-flow mode. Kommineni et al. [18] evaluated ceramic membranes in direct filtration mode for treating Trinity River water and backwash at two pilot plants in the United States, reporting fluxes of 127–455 L/m^2^·day with coagulant addition and turbidity reduction from 23 NTU to below 0.07 NTU. The total organic carbon (TOC) reduction in the filtrate ranged from 20% to 30%. Weiying et al. [19] demonstrated stable operation of a monolith ceramic membrane (0.1 µm) coupled with a coagulation-flocculation-settling tank at a filtration flux of 4 m^3^/m^2^∙day for sand-filter backwash wastewater, with the filtrate meeting drinking water standards for the assessed contaminants, and an average net recovery of 86% of the membrane filtrate.

Al-Tamimi et al. [20] compared a polypropylene (PP, 1 µm) membrane with a ceramic (zirconium, 0.5 µm) membrane for treating Tigris River water and found that the ceramic membrane achieved 99% turbidity removal and >99% Total E. coli removal versus 96% and 99%, respectively, for the PP membrane, although the PP membrane exhibited a higher initial permeate flux. In a study on low-pressure membrane fouling, Gibert et al. [21] investigated a bench-scale MF (polysulfone—PES, 0.2 µm) module treating coagulated and settled surface water, finding that the membrane removed turbidity, Al, and Fe by more than 70%, while TOC and dissolved organic carbon (DOC) were poorly removed (<10%), with foulants being mainly composed of Al and humic substances.

Rasouli et al. [22] employed a hybrid biological ion exchange and gravity-driven membrane process using polymeric and ceramic MF membranes for treating turbid river water, achieving stabilized fluxes of 3.8–5.2 L/m^2^·h and finding that ceramic membranes exhibited higher hydraulically reversible fouling removal than polymeric membranes. Chang et al. [23] provided a comprehensive review of hydraulic backwashing for low-pressure membranes in drinking water treatment, noting that PVDF and PES account for approximately half of the membranes applied, with hollow-fiber configurations being the most common (74.3%), while ceramic (inorganic) membranes represent about 11.6% of the total applications.

Despite these advances, there is limited information on the simultaneous comparison of polymeric and ceramic MF membranes of different configurations (spiral, capillary) for treating backwash water under continuous-flow conditions at operational water treatment facilities, particularly when comparing backwash from both SW and IW water sources. To help bridge this gap, this study evaluates the suitability of three MF membrane modules under pilot-scale continuous flow conditions at two operational water treatment plants in Poland, with the aim of supporting water reuse. Membrane performance was evaluated in terms of contaminant removal (turbidity, dissolved organic carbon, and total number of microorganisms), fouling intensity (based on changes in permeate flux), and treatment cost per cubic meter of backwash water. By employing statistical time-series analysis of permeate flux to characterize the decay of membrane permeability over time, the study provides a reproducible approach to quantify the dynamics of membrane performance. The study aims to determine, under flow-through conditions using real backwash water, the potential for reusing pre-treated backwash water, as well as to compare the costs of the pre-treatment process.

## 2. Materials and Methods

### 2.1. Study Site and Sampling

This study evaluates the effectiveness of MF in treating backwash water from sand filters. Experiments were conducted at two full-scale water treatment plants (WTPs) in Poland, each processing approximately 100,000 cubic meters per day. One treats SW, and the other treats IW [24,25]. Both facilities use sand-bed filtration, which requires periodic backwashing, with the resulting backwash water accounting for approximately 5–7% of the plant’s treated volume [8].

### 2.2. Pre-Treatment, Microfiltration Setup, and Filtration Protocol

**Figure 1:**
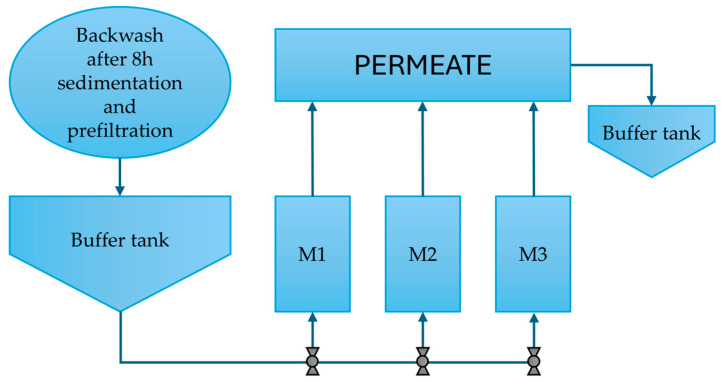
Schematic of the microfiltration installation.

Before MF, the backwash water underwent 8 h of sedimentation, followed by preliminary filtration through a 20 µm bag filter, as described in a previous study [25]. The process was carried out under flow conditions using a pilot-scale system with a nominal capacity of 100 L/h (Figure 1). The system included continuous monitoring of feed flow rate, permeate flux, and transmembrane pressure (TMP).

In the experiments, the performance of three MF modules was evaluated: M1—Spiral with a polymeric (polyvinylidene fluoride—PVDF) membrane, M2—Capillary with a polymeric (polypropylene—PP) membrane, and M3—Capillary with a ceramic (α-Alumina) membrane. All the membranes have a pore size of 0.2 µm. Table 1 presents the technical specifications of each membrane module. All membranes used in the study were supplied by PolymemTech (Warsaw, Poland). The membrane costs used in this study were provided directly by staff from the two water treatment plants where the field campaigns were conducted. These values reflect the approximate purchase prices for individual modules and were used to estimate the cost of treating one cubic meter of backwash water in the membrane efficiency analysis.

Capillary membrane modules were automatically backwashed every 10 min for 30 s. Due to structural constraints, the spiral module could not be backwashed. Each membrane underwent four 7-day filtration cycles (168 h). The first two cycles (C1 and C2 SW) used backwash water from the SW treatment plant, while the following two (C1 and C2 IW) used backwash water from the IW treatment plant. After each cycle, membranes were chemically cleaned with a 2% citric acid solution followed by a 15% sodium hypochlorite solution. All filtration cycles were conducted with a TMP maintained above 2.0 bar and a permeate recovery rate exceeding 75%.

### 2.3. Water Quality Analysis

Turbidity, DOC, and the total number of microorganisms (TNM) were analyzed to evaluate treatment performance. Samples were collected from feed backwash water and permeate at 2, 24, 48, 72, 96, 120, and 144 h of each cycle. Turbidity was measured using a Hach TU5200 turbidimeter. TNM was determined by plate culture using PN-EN ISO 6222:2004 [26], reporting colony-forming units per mL (cfu/mL). DOC was determined using a Shimadzu TOC-L TOC analyzer (Shimadzu, Kyoto, Japan).

### 2.4. Statistical Analysis

Statistical analysis was performed using a Python (v.3.11.7) script developed for this study (provided as Supplementary Materials). The analysis evaluated membrane performance through changes in permeate flux and transmembrane pressure over time. For each filtration cycle, and based on the observed behavior of the data points, a logarithmic regression model of the form:

(1) PF = a·ln(t) +b,

where PF represents permeate flux rate (in L/m^2^∙h), t represents filtration time (in hours), and a (slope) and b (intercept) are model parameters found in the regression.

The permeate flux model was numerically integrated over the interval from hour 1 to 168 and multiplied by the corresponding membrane surface area $M_{i}$ (see Table 1) to estimate total productivity over a one-week filtration cycle. The area under the curve (AUC) value serves as a proxy for the total filtrate volume per cycle (${PF}_{cycle,i}$) in liters, allowing for comparisons across different membrane modules and water types.

(2) $${PF}_{cycle,i}=M_{i}\int_{1}^{168}\left(a\cdot ln(t)+b\right)dt$$

The logarithmic form described in Equation (1) was adopted as an empirical descriptor of flux decline because it captures the characteristic pattern observed during the experiments: a rapid initial decrease followed by a progressively slower decline. Established mechanistic fouling models, such as Hermia’s blocking laws [27,28], assume constant-pressure dead-end filtration with uniform pore geometry and monodisperse foulants, conditions that are not met in the present flow-through system treating heterogeneous backwash water. Moreover, under prolonged operation with real feed water, fouling mechanisms typically shift over time from pore blocking to cake formation, making it difficult for a single mechanistic model to describe the entire cycle. Despite this simplification, the logarithmic form has theoretical backing: Braeken et al. [29] showed that organic adsorption onto membrane pores results in a logarithmic flux–time relationship until adsorption equilibrium is reached, a mechanism consistent with the DOC-rich nature of the backwash water used in this study. Therefore, the logarithmic model is not meant to replace mechanistic fouling analysis but rather to offer a reproducible two-parameter tool for characterizing and comparing flux decline dynamics across different membranes, water types, and filtration cycles.

## 3. Results and Discussion

### 3.1. Backwash Water Quality Characteristics

As shown in Table 2, the feed quality parameters characterizing the backwash water after sedimentation and primary filtration varied across the filtration cycles. The backwash water from SW treatment had lower turbidity than that from IW treatment. This difference was attributed to the presence of iron and manganese compounds in the infiltrated water and in the backwash generated during treatment, with characteristic values reported in previous research [25]. Additionally, it contained fewer microorganisms on average compared to the backwash water from SW treatment, which can be attributed in part to the effects of soil filtration [30]. The variability in backwash water composition was greater with SW treatment, which is associated with seasonal fluctuations in the quality of the abstracted water.

### 3.2. Membrane Performance and Logarithmic Approximation

**Figure 2:**
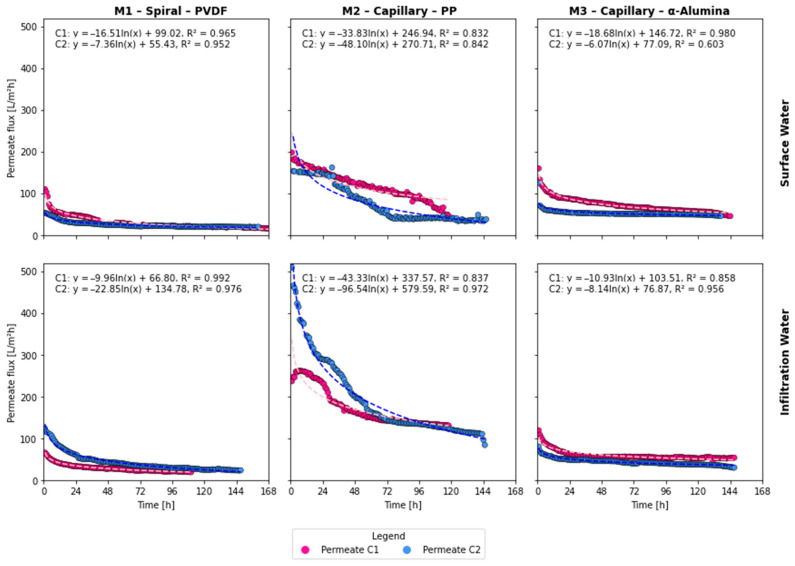
Transport properties for different membranes and cycles. Abbreviations: C1—cycle 1, C2—cycle 2, x—hours since the start of the cycle. Dashed lines correspond to logarithmic regressions.

The time series plots in Figure 2 illustrate the evolution of the transport properties of the membranes, specifically permeate flux, for each module across different cycles and water types, as described in the Section 2.

These time series show the decline in hydraulic performance of the MF membranes over time due to membrane fouling. The intensity of membrane fouling was evaluated using the flux decrease ratio (FDR), defined as:

(3) $$FDR\;=\;\frac{J_{0}-J_{t}}{J_{0}}\times 100\%\;,$$

where $J_{0}$ is the initial membrane flux during the backwash water treatment, and $J_{t}$ is the permeate flux at the end of the process. During MF of SW backwash water, FDR values were 75.04%, 78.97%, and 51.62% for modules M1, M2, and M3, respectively, and 79.84%, 73.89%, and 52.98% for microfiltration of IW backwash water.

Analysis of the FDR results indicates that the largest decline in hydraulic performance occurred in modules M1 and M2, while the lowest decline was observed in module M3. This behavior can be attributed to both the membrane module configuration and the membrane material. Module M1 featured a spiral-wound configuration that precluded membrane backwashing, leading to the accumulation of contaminants on the membrane surface and within its structure throughout the filtration cycle. Module M2 had a capillary configuration that allowed backwashing; however, the membrane was made of polypropylene (PP). According to the literature [31], PP exhibits stronger hydrophobicity compared to α-Alumina and PVDF, which promotes more intense fouling and explains the considerable decline in hydraulic performance observed throughout the filtration cycle. For membranes made of PP, backwashing was insufficient to reduce fouling intensity substantially. In contrast, module M3 showed the smallest decline in permeate flux during the filtration cycle. Ceramic membranes are naturally highly hydrophilic [32], and when combined with backwashing, this property limits the deposition of contaminants on the membrane surface and within its structure.

This decline in permeate flux can be modeled using the logarithmic model in Equation (1). The regression parameters are summarized in Table 3, which also includes the integral of the regression curve from hour 1 to hour 168. This integral, described in Equation (2), estimates the total volume of filtered liquid over the one-week analysis period (${PF}_{cycle,i}$). The fitted models demonstrated high determination coefficients (R^2^ values ranging from 0.60 to 0.99), suggesting a good model agreement in most cases [33]. It is worth noting that the lowest R^2^ (0.60) corresponds to M3-SW-C2, where the ceramic membrane exhibited a nearly constant permeate flux throughout the cycle, as shown in Figure 2. Under these conditions, the logarithmic model, which inherently describes a declining trend, provides a poor fit because there is little flux decline to capture. This behavior suggests that the ceramic membrane reached a quasi-steady state early in the cycle, likely due to reduced fouling potential of the feed water, which, despite a relatively high DOC content, exhibited the lowest turbidity and TNM values of the M3 set, combined with effective periodic backwashing.

**Figure 3:**
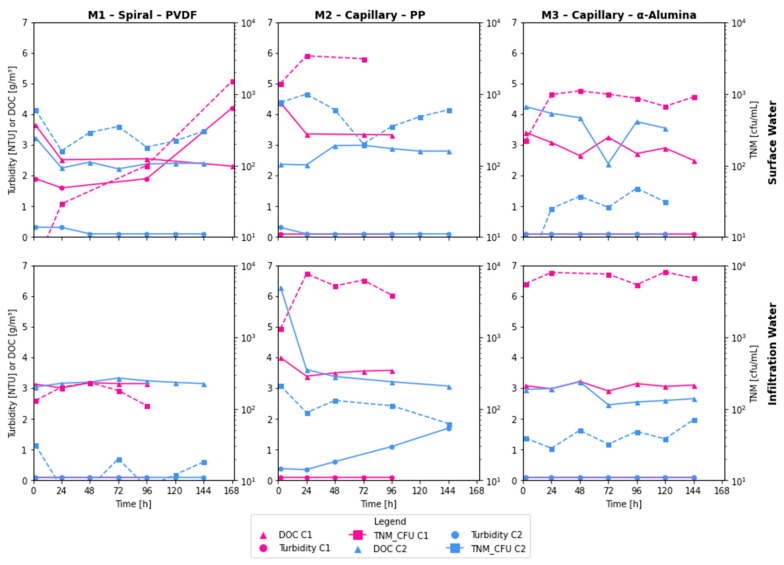
Separation properties of membranes. Abbreviation: C1—cycle 1, C2—cycle 2, DOC—dissolved organic carbon, TNM_CFU—total number of microorganisms (in secondary *Y*-axis).

**Figure 4:**
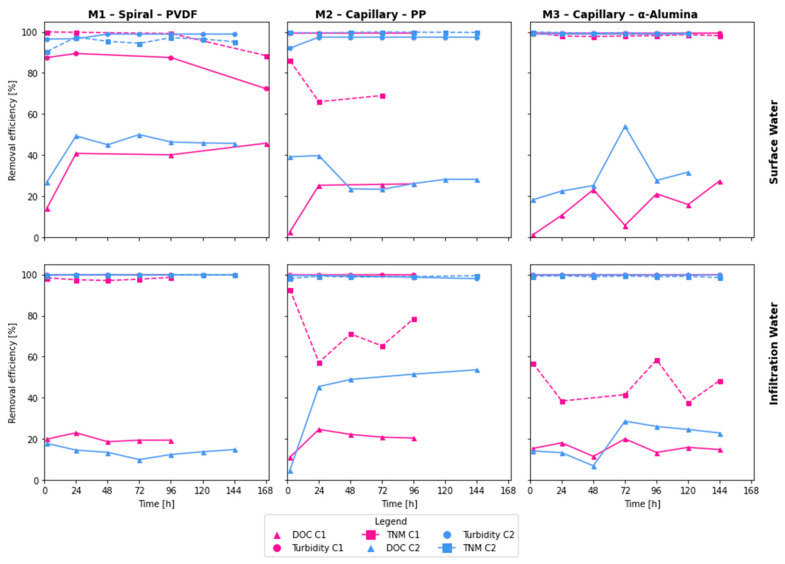
Membranes’ performance in terms of the percentage reduction of the initial concentration. Abbreviation: C1—cycle 1, C2—cycle 2, DOC—dissolved organic carbon, TNM—total number of microorganisms.

As for the pollutants under assessment, Figure 3 and Figure 4, respectively, present the membranes’ performance over time in terms of concentration and percentage of removal regarding the initial concentration. Turbidity removal was consistently high across all membranes, with efficiencies ranging from 97.4% to 99.9% at the end of each cycle, except for M1-SW-C1. Notably, except for M1-SW-C1 and M2-IW-C2, the results largely comply with the EU Directive 2020/2184, which stipulates that turbidity levels should be below 1 NTU [34]. The other two water quality parameters exhibited more variability in their percentage of removal. DOC removal ranged from ~15% to ~55% by the end of the cycles, but absolute DOC in permeate consistently settled around 2–3 g/m^3^, regardless of membrane or source. The achieved efficiencies of organic matter removal were higher than those reported by Park et al. [35] (a maximum reduction of 20%) and were similar to those reported by Wang et al. [36]. Stable TOC content in the permeate is important to maintain the organic matter content in drinking water without undesirable changes [34]. As for TNM removal, it ranged between 37% and over 99%, with second cycles (C2) producing a more stable removal of microorganisms. As shown in the review by Anis et al. [37], many studies confirm the high effectiveness of microorganism removal from water during MF. However, the observed efficiency of 37% is significantly lower than that reported in other studies. Ensuring the high efficiency of microorganism removal from backwash water before its return to the water treatment system is crucial to prevent the regrowth of microorganisms in the treated water, especially pathogenic ones.

The following subsections will provide an assessment of each membrane’s performance, including permeate rates and water quality.

#### 3.2.1. Spiral Membrane Module (M1) with PVDF Membrane Performance

The spiral module M1, equipped with a PVDF membrane, was tested without automatic backwash due to structural constraints, and consistently exhibited high R^2^ values across all four cycles. For the SW cycles, the logarithmic model parameter *a* shows a less steep decline in permeability for cycle 2 (−7.36) than for cycle 1 (−16.51). The change in permeate flow during the second cycle was minimal, with both cycles showing nearly overlapping curves for most of the 168 h. Most of the variation occurred during the initial hours of membrane operation, indicating that after the first chemical cleaning, the membrane’s properties and module performance were restored. Similar findings were reported by Meier-Haack et al. [38]. In the IW cycles, cycle 2 presented the highest initial PF rate, the highest single-cycle volume treated, but also the steepest decrease in permeability across the whole M1 set. The increase in permeate flux may be related to the temperature of the treated backwash water, as discussed by Xu et al. [39]. In the second cycle of MF of backwash water from IW treatment, the initial temperature was higher than that observed at the beginning of the first cycle, suggesting that temperature may be a contributing factor to the increase in permeate flux.

The efficiency of turbidity removal in both cycles of backwash water treatment from IW was close to 100%, except for samples collected during the first two hours of MF. The stable performance of M1 in DOC removal indicates proper membrane operation and is consistent with the commonly reported elimination in this process [20]. In contrast, during the treatment of backwash water from SW, lower DOC removal efficiencies were obtained, with values below 90% in the first cycle and 97–99% in the second cycle. The lower efficiency of DOC removal in SW backwash treatment can be attributed to differences in the composition of the backwash water, particularly the higher content of natural organic matter in the feed backwash water, which may negatively affect turbidity removal [35]. Additionally, the permeate samples from SW backwash in the first cycle had a value of this parameter that consistently exceeded the permissible limit for drinking water of 1 NTU [34]. Besides the presence of organic matter, the reduced efficiency of DOC removal is also influenced by the properties and operational constraints of the PVDF membrane [20], specifically the lack of backwashing and the gradual fouling on its surface.

M1 also effectively eliminated bacteria in backwash water from IW treatment, with removal efficiencies ranging from 97% to 99.9% in both cycles, similar to those reported in previous studies [40]. In the case of backwash water from SW treatment, however, a very high bacterial removal efficiency was initially observed, which gradually decreased, reaching 81% by the end of the second cycle. The decrease in bacterial removal efficiency during the MF process, as observed by Gaveau et al., can be attributed to bacterial cell deformation and their subsequent transport through the membrane [41].

The removal efficiency of organic substances (DOC) from the backwash water of the IW treatment was lower in both MF cycles than in SW. These results may be due to lower levels of inorganic suspended solids in SW, leading to fewer inorganic substances contributing to membrane fouling. In particular, the presence of iron (Fe) and manganese (Mn) compounds in the backwash water from the IW treatment may accelerate membrane fouling. Based on previous measurements taken at the two WTPs [25], the maximum Fe concentrations in raw backwash water from IW (12.11 g/m^3^) were up to 23 times higher than those from SW (0.52 g/m^3^). For Mn, the ratio between maximum concentrations was 2.3 (0.58 g/m^3^ for IW and 0.25 g/m^3^ for SW). The increase in fouling caused by Fe and Mn in ceramic and polymeric membranes was also observed by Dashtban Kenari and Barbeau [42], with the latter having a greater effect.

#### 3.2.2. Capillary Membrane Module (M2) with PP Membrane Performance

Both M2 and M3 were backwashed every 10 min. For SW, the total collected permeate volume in C2 was around 67% of that in C1. In contrast, for IW, the estimated total filtrate volume for C2 reached 14,906 L, among the highest in the entire set.

Regardless of the type of backwash water treated, turbidity reduction was less effective in the second MF cycle. For backwash water from IW treatment, this efficiency, while in the 97–99% range, resulted in the permeate’s turbidity exceeding 1 NTU limits [34]. This result is due to the significantly higher DOC content in the feed backwash water compared to the first cycle and to that found in backwash water from SW treatment. In the second MF cycle of backwash water from SW treatment, the lowest DOC removal efficiency was 92%, with a range of 92% to 97%. The decrease in the effectiveness of this process in the second cycle can be attributed to insufficient removal of suspended solids during backwashing and to the development of biofilm on the membrane surface, which limits the efficiency of turbidity reduction because it is more difficult to remove during backwashing than chemical sludge [43].

In contrast, TNM removal was lower during the first MF cycles, regardless of the type of backwash water treated. At the same time, there was high variability in the efficiencies achieved, due to the simultaneous interactions and influences of multiple factors on microorganism removal during MF. The increase in efficiency and the final TNM count can be attributed to the reduction in pores resulting from the formation of a filter cake on the membrane surface [44], while the increase in penetration may result from pore deformation and microorganism growth [45]. In the second MF cycle for both types of backwash water, a stable and high microorganism removal efficiency was found, similar to that reported in other studies [25,46].

The efficiency of organic substance removal varied widely, and it was generally higher for IW, likely due to the presence of iron compounds in this backwash water and the potential formation of larger-molecular-weight iron-organic complexes that are more easily retained during MF [25]. The increase in this efficiency during the second cycle should be attributed to the higher organic content of the feed backwash water. During the MF of backwash water from SW treatment, a similar average DOC removal efficiency was observed in both cycles.

For both types of backwash water after MF, the DOC concentration ranged from 2.4 to 4.4 gC/m^3^ (except for one sample). The lack of more effective organic substance elimination may be related to the molecular weight distribution, particularly the presence of significant amounts of low-molecular-weight substances that are not effectively removed during MF [25,47].

#### 3.2.3. Capillary α-Alumina Membrane (M3) Performance

Similar to the M2, the ceramic membrane of the M3 module was backwashed every 10 min. For M3, the permeability was higher during the initial cycles for both SW and IW. The absence of permeate flux reduction observed for the membrane in module M3 is due to the durability of ceramic membranes and their resistance to pore deformation [48].

M3 exhibited high turbidity removal across all cycles, with values exceeding 99% at all data points after 24 h, indicating the technology’s adequacy for this water quality parameter. Consistently high turbidity removal efficiency during MF using a ceramic membrane has also been reported in previous studies [35,49] across multiple successive MF cycles. The same study reported variations in microorganism removal efficiency, attributed to changes in water composition and membrane fouling.

During the first MF cycle for IW, there was high variability in microorganism removal efficiency. This likely occurs because the filter cake stabilizes on the membrane surface [44]. In the C2 of backwash water from the IW treatment, this efficiency remained high (>98.5%). Similarly, in C2 conducted for backwash water from SW treatment, a stable and very high (>99.2%) efficiency of microorganism removal was maintained.

The level of microorganism retention achieved for both types of backwash water resulted in TNM counts in the permeate that allowed their return to the water treatment systems, and these counts were significantly lower than those in the source water. In contrast, the retention of organic substances was considerably lower than that for turbidity and TNM. The variability in DOC removal efficiency is due to differences in the properties of these substances, and the DOC concentration in the permeate most likely results from the presence of low-molecular-weight substances in the backwash water.

### 3.3. Additional Remarks

A high count of psychrophilic bacteria is a water quality parameter that prevents the introduction of pre-treated backwash water into the basic technological system of water treatment after the filtration process. The average number of microorganisms in the pre-treated backwash water was lowest in the water treated with the spiral membrane (M1) (Figure 3), allowing for the reuse of this backwash water after filtration in both SW and IW. For membrane M3, it is possible to redirect the pre-treated backwash water to the beginning of the SW treatment system. However, membrane M2 does not offer this possibility, as the number of microorganisms in its permeate is comparable to or even higher than that in the feed water (see Table 2), with a similar observation noted for the first cycle with IW and membrane M3.

The effectiveness of DOC removal varied significantly between membranes and depended on the type of backwash water. The highest removal efficiency for both backwash types was observed with M1, likely due to the absence of backflushing and the formation of a filtration layer on its surface. The presence of organic substances and their retention on the membrane surface significantly contribute to membrane fouling and a reduction in permeate flux. Therefore, regular backwashing reduces the fouling phenomenon while simultaneously decreasing the effectiveness of DOC removal [50].

### 3.4. Cost Analysis

**Figure 5:**
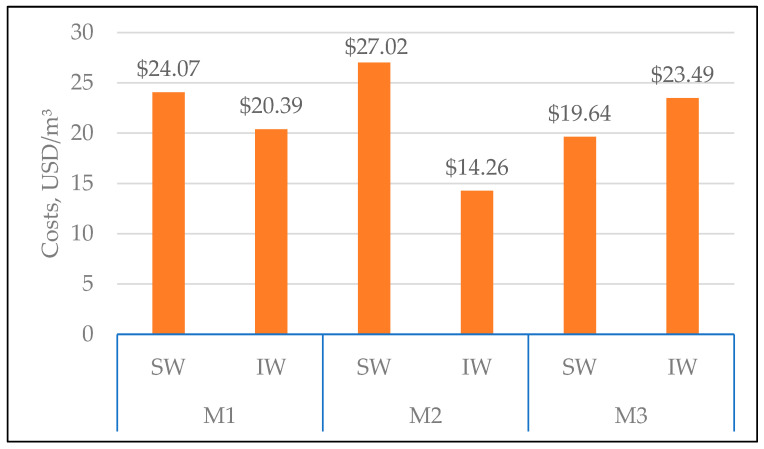
Average costs of backwash water treatment, considering a lifespan of two cycles.

This subsection provides a simplified cost assessment based on the volume of permeate produced by each membrane during MF, and the results are summarized in Figure 5. The analysis considers only membrane costs, excluding electricity and cleaning chemical expenses. This approach assumes that operational costs are similar across membrane types and do not influence comparative outcomes. Due to differences in backwash water contamination between facilities, the assessment treats each membrane and washing type separately.

The cost calculations assume a membrane lifespan of two filtration cycles, which is a major simplification, as the typical lifespan of polymeric membranes is 3–5 years [16] and up to 10 years for ceramic membranes [51]. This oversimplification explains why the obtained costs are significantly higher than those reported by Pham et al. [52], despite their inclusion of installation and purchase costs, as well as operational expenses. Additionally, they adopted a cost recovery of $30–60/m^2^. Since the membranes used in the present work maintained their properties and could be utilized for an extended period, the purpose of Figure 4 is to provide a standard for comparing the results specific to the test conditions used in this study. This limitation may not directly reflect full-scale operational scenarios, where factors such as fouling dynamics, cleaning frequency, and membrane replacement schedules can significantly influence overall costs.

The cost of treating one cubic meter of backwash water was considerably higher for SW than for IW for membranes M1 and M2 (Figure 5), especially for M2, which was almost twice as expensive for SW compared to IW. This membrane exhibited the highest variation in the obtained permeate flux, which can be partially attributed to differences in the temperature of the feed backwash water used with this membrane [39]. For M3, the treatment cost was about 20% higher for IW than for SW.

### 3.5. Benchmarking Against Existing Literature

To provide some context regarding the performance of the three MF modules, Table 4 compares these results with studies on MF treatment of spent filter backwash water and related recycle streams in drinking water treatment plants. The comparison focuses on permeate flux, turbidity removal, organic matter removal (DOC/TOC), and, where available, microbiological indicators.

The PP capillary membrane (M2) showed the highest initial fluxes among the tested modules, with intercept values ranging from about 247 to 580 L/m^2^·h and final values of 24–116 L/m^2^·h after 168 h of continuous operation (Table 3). These values are comparable to or higher than the cross-flow fluxes of 102–280 L/m^2^·h reported by Mueller et al. [17] for ceramic MF and UF membranes treating spent filter backwash water from a dam water treatment plant. Conversely, the ceramic capillary membrane (M3) exhibited initial fluxes of 77–147 L/m^2^·h and final fluxes of 35–51 L/m^2^·h, indicating more moderate but stable hydraulic performance, consistent with the durability reported for ceramic membranes in similar applications. The PVDF spiral membrane (M1), operated without backwash, showed lower final fluxes of 14–18 L/m^2^·h, reflecting its greater vulnerability to irreversible fouling.

All three modules achieved high turbidity removal. In this study, turbidity reduction ranged from 90–99.9%, with ceramic membrane M3 consistently exceeding 99% after the first 24 h of filtration. These values are comparable to those reported by Kommineni et al. [18], who achieved >99.7% turbidity removal (from 23 NTU to <0.07 NTU) using monolithic ceramic membranes treating river water and filter backwash with coagulant addition, and by Mueller et al. [17], who obtained permeate particle counts below 100 mL^−1^ when treating spent filter backwash water. In the present study, similar turbidity control was achieved without coagulant dosing, relying only on sedimentation, 20 µm bag filtration, and MF.

Organic matter removal in this work (approximately 15–55% DOC, with permeate DOC typically 2–3 gC/m^3^) is comparable to or higher than the TOC reduction of 20–30% reported by Kommineni et al. [18] and the ~50% DOC removal obtained by Mueller et al. [17] for ceramic membranes treating backwash water. The highest DOC removal was associated with the PVDF spiral membrane (M1) for SW backwash and with the PP capillary membrane (M2) for IW backwash. This behavior can be linked to the formation of a filtration cake on M1 that was not backwashed, and to the presence of iron–organic complexes in the IW backwash that MF more easily retains.

Microbiological data for spent filter backwash treatment are scarce in the literature; most studies focus on turbidity and bulk chemistry [17,18,19]. In this context, the present study adds detail by quantifying the total number of microorganisms (TNM) in feed and permeate. The PVDF spiral membrane (M1) achieved the highest median TNM removal (97%), followed by M2 (84%) and M3 (70% in the first cycles but >98.5% in the second cycles). As previously mentioned, the improvement in TNM removal between cycles for M2 and M3 suggests that the development of a stable cake layer enhances microbial retention [44]. Taken together, these results indicate that the three MF modules tested here perform at least comparably to ceramic MF systems previously reported for spent filter backwash, while providing a broader assessment that includes polymeric and ceramic materials, two source waters (SW and IW), and a simplified cost analysis.

## 4. Conclusions

This study assessed the performance of three membrane modules: M1—Spiral with a PVDF membrane, M2—Capillary with a PP membrane, and M3—Capillary with an α-Alumina membrane, in the MF pre-treatment of backwash water from surface water (SW) and infiltration water (IW) purification plants. Mathematical regression was used to develop logarithmic models describing permeate flux decline for all membranes tested. These models support early-stage performance prediction in short-term filtration processes. Among the main findings, no consistent trend in flux decline was observed across filtration cycles, mainly due to bacterial load and other characteristics of the feed backwash water, which emerged as limiting factors for membrane throughput.

The highest permeate flux rates (in L/m^2^∙h) were observed for M2, regardless of water type or operational conditions, suggesting a more stable hydraulic performance under varying settings, despite indications that the microorganism content in its permeate may be similar to or exceed that of the feed backwash water. Within this context, none of the membranes achieved complete disinfection, but M1 provided the most effective reduction in microorganisms and organic matter for both water sources. The membrane module M3 showed the greatest variability in both hydraulic performance and removal efficiency, indicating that it heavily depends on the nature of the backwash water treated. Regarding the treatment costs per cubic meter comparison based on experimental data and assumptions, these were usually higher for SW than for IW.

These findings provide a foundation for optimizing membrane selection and operational strategies to reuse backwash water. However, the simplified assumptions, particularly regarding membrane lifespan and cleaning procedures, limit the direct extrapolation to full-scale systems. The logarithmic model provided a practical framework for comparing flux decline across membranes, water types, and filtration cycles; however, its empirical nature and the lower fit observed for certain membrane-cycle combinations (e.g., M3-SW-C2, R^2^ = 0.60) indicate that future studies should explore mechanistic or hybrid fouling models to better capture flux variability under fluctuating feed water conditions.

Future research should incorporate longer filtration series to allow for a more comprehensive evaluation of membrane aging and fouling patterns. Capturing SEM images would help clarify changes in membrane pore structure. At the same time, the inclusion of additional performance indicators, such as organic matter content and particle-size distributions, can provide insights into hydraulic behavior and water-quality outcomes. Extending the number of filtration cycles per membrane is crucial for more accurately simulating real-world usage. Finally, evaluating cleaning effectiveness and recovery efficiency will support more accurate estimates of operational costs.

## Figures and Tables

**Table 1:** Characteristics of the membranes and membrane modules used.

Membrane Module	Type	Material	Membrane Surface (m^2^)	Maximum Temperature (°C)	Maximum Pressure (MPa)	Unit Price * (USD)
M1	Spiral	PVDF	1.80	45	1.0	400
M2	Capillary	PP	0.50	45	0.3	400
M3	Capillary	α-Alumina	1.26	140	0.8	500

* Price in the year 2023 for the pilot-scale membrane modules.

**Table 2:** Characteristics of feed backwash water. Abbreviations: SW—surface water, IW—infiltration water, TNM—total number of microorganisms, DOC—dissolved organic carbon.

Membrane	Source	Cycle	Turbidity (NTU)	TNM (cfu/mL)	DOC (g/m^3^)	Temperature (°C)
M1	SW	1	15.20	13,000	4.26	10.3
		2	9.20	6300	4.44	10.5
	IW	1	41.00	7900	3.91	11.7
		2	66.00	6900	3.70	12.2
M2	SW	1	12.60	10,000	4.50	10.8
		2	3.90	210,000	3.90	11.0
	IW	1	64.00	18,000	4.50	11.8
		2	84.00	11,000	6.62	12.5
M3	SW	1	22.30	49,000	3.45	10.6
		2	11.70	6200	5.20	10.9
	IW	1	92.00	13,000	3.64	11.3
		2	74.00	4700	3.45	11.8

**Table 3:** Characteristics of the membrane modules used.

Membrane	Source	Cycle	*a*—Slope	*b*—Intercept	R^2^	*PF_cycle,i_* (L)	Total *PF_source,i_* * (L)
M1	SW	1	−16.51	99.02	0.97	9143	16,617
		2	−7.36	55.43	0.95	7474	
	IW	1	−9.96	66.80	0.99	7643	19,615
		2	−22.85	134.78	0.98	11,972	
M2	SW	1	−33.83	246.94	0.83	8885	14,804
		2	−48.10	270.71	0.84	5919	
	IW	1	−43.33	337.57	0.84	13,153	28,059
		2	−96.54	579.59	0.97	14,906	
M3	SW	1	−18.68	146.72	0.98	14,543	25,457
		2	−6.07	77.09	0.60	10,914	
	IW	1	−10.93	103.51	0.86	12,223	21,285
		2	−8.14	76.87	0.96	9062	

* total estimated volume of permeate obtained from cycles 1 and 2. Cycle duration assumed: 168 h.

**Table 4:** Selected studies on MF treatment of spent filter backwash water and recycle streams, compared with the three membrane modules evaluated in this study.

Study	Membrane Material/Configuration	Pore Size (µm)	Feed Water	Permeate Flux (L/m^2^·h)	Turbidity Removal (%)	DOC/TOC Removal (%)	Microorganism Removal
Present study (M1)	PVDF/spiral	0.2	Sand filter BW (SW & IW)	55–135 (initial); 14–18 (at 168 h)	52–99.8	~10–50 (DOC)	TNM: median 98.61%
Present study (M2)	PP/capillary	0.2	Sand filter BW (SW & IW)	247–580 (initial); 24–116 (at 168 h)	92–99.8	~3–54 (DOC)	TNM: median 98.81%
Present study (M3)	α-Alumina/capillary	0.2	Sand filter BW (SW & IW)	77–147 (initial); 35–51 (at 168 h)	>99.1	~3–54 (DOC)	TNM: median > 97.86%
Mueller et al. [17]	α-Al_2_O_3_; SiC/tubular	0.2; 0.5	Spent filter BW (dam water)	102–280 (cross-flow)	Particle counts: New 10–1000/mL Prefouled <100/mL	Up to 57 (DOC)	n.r.
Kommineni et al. [18]	Ceramic/monolith	n.s.	River water; spent filter BW	127–455 (75–268 gal/ft^2^·d)	>99.7	20–30 (TOC)	n.r.
Weiying et al. [19]	Monolith ceramic/tubular	0.1	BW from rapid sand filter	83–166 (2 to 4 m^3^/m^2^·d)	n.r.	n.r.	Undetected in the filtrate

Abbreviations: BW—backwash water; SW—surface water; IW—infiltration water; n.r.—not reported; n.s.—not specified.

## Data Availability

The original contributions presented in this study are included in the article and Supplementary Materials. For any further inquiries, please contact the corresponding authors.

## Supplementary Materials

The following supporting information can be downloaded at: https://www.mdpi.com/article/10.3390/membranes16050169/s1, The Python 3.11.7 code and datasets supporting this study are provided as Supplementary Materials. WQdataMF202508.csv: this is a CSV with the experimental dataset employed in the python script; WQdataMF202508_t0added.xlsx: this is a XLSX file with the experimental dataset and the initial conditions also included for each cycle and type of water; MFscript202508_m2v1.py: this is the Python script employed to process and analyze the datasets and create the plots presented in this manuscript).

## References

[1] Mannina G., Gulhan H., Ni B.-J. (2022). Water Reuse from Wastewater Treatment: The Transition towards Circular Economy in the Water Sector. Bioresour. Technol..

[2] Sauvé S., Lamontagne S., Dupras J., Stahel W. (2021). Circular Economy of Water: Tackling Quantity, Quality and Footprint of Water. Environ. Dev..

[3] Hu Z., Chen B., Chen W., Tan D., Shen D. (2021). Review of Model-Based and Data-Driven Approaches for Leak Detection and Location in Water Distribution Systems. Water Supply.

[4] Beshr S., Moustafa M., Fayed M., Aly S. (2023). Evaluation of Water Consumption in Rapid Sand Filters Backwashed under Varied Physical Conditions. Alex. Eng. J..

[5] Wolska M., Urbańska-Kozłowska H., Solipiwko-Pieścik A. (2025). An Assessment of Coagulation Process Efficiency as a Pre-Treatment for Reusing Filtration Backwash in Water Treatment Plants. Arch. Civ. Eng..

[6] Zielina M., Dąbrowski W. (2021). Energy and Water Savings during Backwashing of Rapid Filter Plants. Energies.

[7] Abdel-Shafy H.I., El-Khateeb M.A., Mansour M.S.M., Salem M.A., Abdel-Shafy N.H. (2020). Innovative System for Recycling of Backwashing Water in Drinking Water Plant. Egypt. J. Chem..

[8] Wolska M., Solipiwko-Pieścik A., Urbańska-Kozłowska H. (2024). Disinfection as a Stabilization Method for Backwash Water Reuse. Desalin. Water Treat..

[9] Abdiyev K., Azat S., Kuldeyev E., Ybyraiymkul D., Kabdrakhmanova S., Berndtsson R., Khalkhabai B., Kabdrakhmanova A., Sultakhan S. (2023). Review of Slow Sand Filtration for Raw Water Treatment with Potential Application in Less-Developed Countries. Water.

[10] Xie T., Xu Y., Liu X., Jiang C., Liang H., Liu S., Du H., Li S., Dong H., Qiang Z. (2024). Microbial Safety Evaluation for Recycling of Sand-Filter Backwash Water in a Water Plant in Southern China. J. Water Process Eng..

[11] Komorowska-Kaufman M., Toczek M. (2022). Recirculation of Backwash Water in the Water Treatment Plant for the Needs of the Combined Heat and Power Plant. J. Ecol. Eng..

[12] Sangrola S., Kumar A., Nivedhitha S., Chatterjee J., Subbiah S., Narayanasamy S. (2020). Optimization of Backwash Parameters for Hollow Fiber Membrane Filters Used for Water Purification. J. Water Supply Res. Technol..

[13] Wu Y., Wang H., Zhu J. (2022). Influence of Reclaimed Water Quality on Infiltration Characteristics of Typical Subtropical Zone Soils: A Case Study in South China. Sustainability.

[14] Basset C., Abou Najm M., Ghezzehei T., Hao X., Daccache A. (2023). How Does Soil Structure Affect Water Infiltration? A Meta-Data Systematic Review. Soil Tillage Res..

[15] Valhondo C., Martínez-Landa L., Carrera J., Díaz-Cruz S.M., Amalfitano S., Levantesi C. (2020). Six Artificial Recharge Pilot Replicates to Gain Insight into Water Quality Enhancement Processes. Chemosphere.

[16] Cogan N.G., Ozturk D., Ishida K., Safarik J., Chellam S. (2022). Membrane Aging Effects on Water Recovery during Full-Scale Potable Reuse: Mathematical Optimization of Backwashing Frequency for Constant-Flux Microfiltration. Sep. Purif. Technol..

[17] Mueller U., Biwer G., Baldauf G. (2010). Ceramic Membranes for Water Treatment. Water Sci. Technol. Water Supply.

[18] Kommineni S.N., Bryck J., Stringer C., Stevens C., Meyers N., Karnik B., Hoffman R., Sullivan L. (2010). Evaluation of an Emerging Water Treatment Technology: Ceramic Membranes. Water Sci. Technol. Water Supply.

[19] Weiying L., Yuasa A., Bingzhi D., Huiping D., Naiyun G. (2010). Study on Backwash Wastewater from Rapid Sand-Filter by Monolith Ceramic Membrane. Desalination.

[20] Al-Tamimi N.J., Al-Alawy A.F., Al-Shaeli M. (2025). Evaluation of Microfiltration and Ultrafiltration Membranes for Improving Water Quality: Removal of Turbidity, Suspended Solids, and Bacteria from the Tigris River. Iraqi J. Chem. Pet. Eng..

[21] Gibert O., Lefèvre B., Ferrer O., Prats G., Bernat X., Paraira M. (2016). Composition and Reversibility of Fouling on Low-Pressure Membranes in the Filtration of Coagulated Water: Insights into Organic Fractions Behaviour. Desalin. Water Treat..

[22] Rasouli Y., Barbeau B., Maltais-Tariant R., Boudoux C., Claveau-Mallet D. (2024). Impact of Cleaning on Membrane Performance during Surface Water Treatment: A Hybrid Process with Biological Ion Exchange and Gravity-Driven Membranes. Membranes.

[23] Chang H., Liang H., Qu F., Liu B., Yu H., Du X., Li G., Snyder S.A. (2017). Hydraulic Backwashing for Low-Pressure Membranes in Drinking Water Treatment: A Review. J. Memb. Sci..

[24] Wolska M., Urbańska-Kozłowska H. (2023). Assessing the Possibilities of Backwash Water Reuse Filters in the Water Treatment System—Case Analysis. Water.

[25] Wolska M., Kabsch-Korbutowicz M., Rosińska A., Solipiwko-Pieścik A., Urbańska-Kozłowska H. (2024). The Use of Microfiltration for the Pretreatment of Backwash Water from Sand Filters. Materials.

[26] (2004). Water Quality—Enumeration of Culturable Microorganisms—Colony Count by Inoculation in a Nutrient Agar Culture Medium.

[27] Hu G., Wang Z. (2025). A Review of Mathematical Models in the Microfiltration Membrane Process. J. Water Process Eng..

[28] Pereira G.L.D., Cardozo-Filho L., Jegatheesan V., Guirardello R. (2023). Generalization and Expansion of the Hermia Model for a Better Understanding of Membrane Fouling. Membranes.

[29] Braeken L., Van Der Bruggen B., Vandecasteele C. (2006). Flux Decline in Nanofiltration Due to Adsorption of Dissolved Organic Compounds: Model Prediction of Time Dependency. J. Phys. Chem. B.

[30] Abu-Ashour J., Joy D.M., Lee H., Whiteley H.R., Zelin S. (1994). Transport of Microorganisms through Soil. Water Air Soil Pollut..

[31] Ariono D., Wardani A.K. (2017). Modification and Applications of Hydrophilic Polypropylene Membrane. IOP Conf. Ser. Mater. Sci. Eng..

[32] Chen M., Heijman S.G.J., Rietveld L.C. (2021). State-of-the-Art Ceramic Membranes for Oily Wastewater Treatment: Modification and Application. Membranes.

[33] Santhi C., Arnold J.G., Williams J.R., Dugas W.A., Srinivasan R., Hauck L.M. (2001). Validation of the SWAT Model on a Large River Basin with Point and Nonpoint Sources. JAWRA J. Am. Water Resour. Assoc..

[34] (2020). Directive (EU) 2020/2184 of the European Parliament and of the Council of 16 December 2020 on the Quality of Water Intended for Human Consumption (Recast). Off. J. Eur. Union.

[35] Park W., Jeong S., Im S.-J., Jang A. (2020). High Turbidity Water Treatment by Ceramic Microfiltration Membrane: Fouling Identification and Process Optimization. Environ. Technol. Innov..

[36] Wang Y., Fortunato L., Jeong S., Leiknes T. (2017). Gravity-Driven Membrane System for Secondary Wastewater Effluent Treatment: Filtration Performance and Fouling Characterization. Sep. Purif. Technol..

[37] Anis S.F., Hashaikeh R., Hilal N. (2019). Microfiltration Membrane Processes: A Review of Research Trends over the Past Decade. J. Water Process Eng..

[38] Meier-Haack J., Booker N., Carroll T. (2003). A Permeability-Controlled Microfiltration Membrane for Reduced Fouling in Drinking Water Treatment. Water Res..

[39] Xu B., Gao W., Liao B., Bai H., Qiao Y., Turek W. (2023). A Review of Temperature Effects on Membrane Filtration. Membranes.

[40] Helling A., Grote C., Büning D., Ulbricht M., Wessling M., Polakovic M., Thom V. (2019). Influence of Flow Alterations on Bacteria Retention during Microfiltration. J. Memb. Sci..

[41] Gaveau A., Coetsier C., Roques C., Bacchin P., Dague E., Causserand C. (2017). Bacteria Transfer by Deformation through Microfiltration Membrane. J. Memb. Sci..

[42] Dashtban Kenari S.L., Barbeau B. (2016). Understanding Ultrafiltration Fouling of Ceramic and Polymeric Membranes Caused by Oxidized Iron and Manganese in Water Treatment. J. Memb. Sci..

[43] Gul A., Hruza J., Yalcinkaya F. (2021). Fouling and Chemical Cleaning of Microfiltration Membranes: A Mini-Review. Polymers.

[44] Goswami K.P., Pugazhenthi G. (2020). Credibility of Polymeric and Ceramic Membrane Filtration in the Removal of Bacteria and Virus from Water: A Review. J. Environ. Manage..

[45] Luongo V., Mattei M.R., Frunzo L., D’Acunto B., Gupta K., Chellam S., Cogan N. (2022). A Transient Biological Fouling Model for Constant Flux Microfiltration. Math. Biosci. Eng..

[46] Daneluz J., da Silva G.F., Duarte J., Turossi T.C., dos Santos V., Baldasso C., Daneluz A.C. (2023). Membrane Separation Process of Microfiltration Applied to the Filtration of Kombuchas. Food Chem. Adv..

[47] Devaisy S., Kandasamy J., Nguyen T.V., Johir M.A.H., Ratnaweera H., Vigneswaran S. (2022). Comparison of Membrane-Based Treatment Methods for the Removal of Micro-Pollutants from Reclaimed Water. Water.

[48] Matos M., Suárez M.A., Gutiérrez G., Coca J., Pazos C. (2013). Emulsification with Microfiltration Ceramic Membranes: A Different Approach to Droplet Formation Mechanism. J. Memb. Sci..

[49] Hakami M.W., Alkhudhiri A., Al-Batty S., Zacharof M.-P., Maddy J., Hilal N. (2020). Ceramic Microfiltration Membranes in Wastewater Treatment: Filtration Behavior, Fouling and Prevention. Membranes.

[50] Lin C.-F., Yu-Chen Lin A., Sri Chandana P., Tsai C.-Y. (2009). Effects of Mass Retention of Dissolved Organic Matter and Membrane Pore Size on Membrane Fouling and Flux Decline. Water Res..

[51] Omar N.M.A., Othman M.H.D., Tai Z.S., Kurniawan T.A., Puteh M.H., Jaafar J., Rahman M.A., Ismail A.F., Rajamohan N., Abdullah H. (2024). Recent Strategies for Enhancing the Performance and Lifespan of Low-Cost Ceramic Membranes in Water Filtration and Treatment Processes: A Review. J. Water Process Eng..

[52] Pham D.C., Cao T.M.D., Nguyen M.C., Nguyen T.D., Nguyen V.H., Bui V.H., Nguyen T.T.T. (2022). Integrating Photocatalysis and Microfiltration for Methylene Blue Degradation: Kinetics and Cost Estimation. Chem. Eng. Technol..

